# Pharmacological and Toxicological Properties of the Potent Oral *γ*-Secretase Modulator BPN-15606[Fn FN4]

**DOI:** 10.1124/jpet.117.240861

**Published:** 2017-07

**Authors:** Steven L. Wagner, Kevin D. Rynearson, Steven K. Duddy, Can Zhang, Phuong D. Nguyen, Ann Becker, Uyen Vo, Deborah Masliah, Louise Monte, Justin B. Klee, Corinne M. Echmalian, Weiming Xia, Luisa Quinti, Graham Johnson, Jiunn H. Lin, Doo Y. Kim, William C. Mobley, Robert A. Rissman, Rudolph E. Tanzi

**Affiliations:** Department of Neurosciences, University of California, San Diego, La Jolla, California (S.L.W., K.D.R., P.D.N., A.B., U.V., D.M., L.M., W.C.M., R.A.R.); Integrated Nonclinical Development Solutions, Ann Arbor, Michigan (S.K.D.); NuPharmAdvise, Sanbornton, New Hampshire (G.J.); Biopharm Consulting Partners, Ambler, Pennsylvania (J.H.L.); and Genetics and Aging Research Unit, Department of Neurology, Massachusetts General Hospital, Charlestown, Massachusetts (C.Z., J.B.K., C.M.E., W.X., L.Q., D.Y.K., R.E.T.)

## Abstract

Alzheimer’s disease (AD) is characterized neuropathologically by an abundance of 1) neuritic plaques, which are primarily composed of a fibrillar 42-amino-acid amyloid-*β* peptide (A*β*), as well as 2) neurofibrillary tangles composed of aggregates of hyperphosporylated tau. Elevations in the concentrations of the A*β*42 peptide in the brain, as a result of either increased production or decreased clearance, are postulated to initiate and drive the AD pathologic process. We initially introduced a novel class of bridged aromatics referred t*γ*-secretase modulatoro as *γ*-secretase modulators that inhibited the production of the A*β*42 peptide and to a lesser degree the A*β*40 peptide while concomitantly increasing the production of the carboxyl-truncated A*β*38 and A*β*37 peptides. These modulators potently lower A*β*42 levels without inhibiting the *γ*-secretase–mediated proteolysis of Notch or causing accumulation of carboxyl-terminal fragments of APP. In this study, we report a large number of pharmacological studies and early assessment of toxicology characterizing a highly potent *γ*-secretase modulator (GSM), (*S*)-*N*-(1-(4-fluorophenyl)ethyl)-6-(6-methoxy-5-(4-methyl-1*H*-imidazol-1-yl)pyridin-2-yl)-4-methylpyridazin-3-amine (BPN-15606). BPN-15606 displayed the ability to significantly lower A*β*42 levels in the central nervous system of rats and mice at doses as low as 5–10 mg/kg, significantly reduce A*β* neuritic plaque load in an AD transgenic mouse model, and significantly reduce levels of insoluble A*β*42 and pThr181 tau in a three-dimensional human neural cell culture model. Results from repeat-dose toxicity studies in rats and dose escalation/repeat-dose toxicity studies in nonhuman primates have designated this GSM for 28-day Investigational New Drug-enabling good laboratory practice studies and positioned it as a candidate for human clinical trials.

## Introduction

Alzheimer’s disease (AD) is pathologically characterized by neuritic plaques and neurofibrillary tangles that result in a significant loss of neurons and synapses in areas of the brain important for cognition (Tanzi and Bertram, 2005). AD is an emerging health crisis that imposes a severe economic burden on those affected. In the absence of any disease-modifying treatments, the costs of AD may bankrupt our healthcare system in the next three decades (http://www.Alz.Org). Unfortunately, existing treatments are merely palliative, providing only temporary symptomatic benefit, and do not affect the underlying progression of the disease.

A number of potential disease-modifying therapeutic approaches for AD either have previously failed or are currently just beginning to be tested clinically, yet none to date have been shown to impact disease progression (Qiu et al., 2009; Sabbagh and Cummings, 2011). Most treatment strategies currently being pursued have arisen from the genetic, biochemical, and morphologic data implicating the amyloid precursor protein (APP) and the amyloid-*β* peptide (A*β*) products from proteolytic processing in the pathogenesis of AD (Toyn et al., 2014). Consequentially, therapeutic strategies have focused on inhibiting the enzymes (secretases) responsible for A*β* production, thereby reducing the levels of all A*β* peptide species. Although *β*-site amyloid precursor protein cleaving enzyme (BACE) inhibitors are currently under clinical scrutiny (Portelius et al., 2014), *γ*-secretase inhibitors have not demonstrated efficacy and have shown significant side effects (Fleisher et al., 2008; Coric et al., 2012). In contrast, immunotherapy approaches that aim at clearing deposited A*β* peptides have recently shown potential against AD; however, this is only after years of failures (Gilman et al., 2005; Miles et al., 2013; Salloway et al., 2014). Biogen recently reported results of a 52-week, 166-patient phase 1b clinical trial with an anti-A*β* human monoclonal antibody, aducanumab, which showed a statistically significant dose-dependent reduction of amyloid plaques as measured by florbetapir positron emission tomography imaging, and statistically significant reduction in cognitive decline as measured by Mini-Mental State Examination and Clinical Dementia Rating, relative to placebo, lending significant support to therapeutic strategies targeting plaque-associated A*β* peptides for the treatment of AD (Sevigny et al., 2016).

The neuritic plaques associated with AD are composed predominantly of A*β*42 (Iwatsubo et al., 1994), and the most common biochemical phenotype observed for the more than 200 different familial AD–linked mutations is an increased ratio of A*β*42/A*β*40 (Kumar-Singh et al., 2006; Potter et al., 2013). Moreover, a large body of data points to A*β*42 as the most potently pathogenic of this family of peptides (Tanzi and Bertram, 2005). Thus, selective attenuation of A*β*42 relative to the shorter A*β* peptides (i.e., A*β*40, A*β*38, and A*β*37) may prove to be a novel and efficacious avenue for interrupting AD progression (Kounnas et al., 2010; Wagner et al., 2012, 2014). Because the A*β* peptides are produced as the result of the sequential proteolytic processing of APP by the aspartyl protease known as *γ* -secretase, small molecules capable of modulating the enzyme have been intensely pursued.

Our therapeutic approach is based on the amyloid cascade hypothesis (Hardy and Higgins, 1992) and utilizes a small molecule *γ* -secretase modulator to selectively attenuate the production of A*β*42 while increasing the production of truncated A*β* species. Whereas previous therapeutic approaches utilizing small molecules targeting the amyloid cascade hypothesis have been shown to be toxic (e.g., *γ*-secretase inhibitors such as semagacestat and avagacestat), *γ*-secretase modulation addresses the oligomeric-prone A*β*42 peptide and does not interfere with overall enzyme function, thereby avoiding the inhibitor-related side effects. We view the *γ*-secretase modulation approach as superior to the use of vaccines and passive immunization that require invasive procedures and immune regulation. In addition, because *γ*-secretase modulators (GSMs) directly attenuate the level of A*β*42 and to a lesser degree A*β*40, while increasing levels of shorter A*β* peptides (A*β*38 and A*β*37), they may prove to be easier to test, evaluate, and monitor clinically than compounds that inhibit the activities of either *β*-secretase or *γ*-secretase.

Beyond slowing progression in symptomatic AD, an important additional therapeutic goal of disease-modifying approaches is to prevent AD in high-risk populations (Golde et al., 2011). This objective will be viewed as increasingly important if current immunotherapy and BACE inhibitor trials reinforce evidence that intervening after the development of abundant pathology severely limits efficacy. Intervening in AD for prevention or at stages of earliest symptoms requires many years of treatment, and a high priority is to identify drugs that are more affordable than antibodies, and that can be delivered orally, safely, and for decades before the onset of AD in people at risk by virtue of genetic predisposition, risk factors, or biomarker related to presymptomatic pathology.

## Materials and Methods

### Compounds

The novel GSM (*S*)-*N*-(1-(4-fluorophenyl)ethyl)-6-(6-methoxy-5-(4-methyl-1*H*-imidazol-1-yl)pyridin-2-yl)-4-methylpyridazin-3-amine (BPN-15606) was synthesized at Albany Molecular Research Institute (Albany, NY), using the methods reported in the University of California, San Diego patent (Wagner et al., 2016). The *γ*-secretase inhibitor ***N*-[*N*-**(3,5-difluorophenacetyl)-*L*-alanyl]-*S*-phenylglycine t-butyl ester was purchased from Sigma-Aldrich (St. Louis, MO).

### A*β* Enzyme-Linked Immunosorbent Assays

All A*β* assays, including the anti-A*β* immunoprecipitation/matrix-assisted laser desorption/ionization time of flight, were carried out as described previously (Wagner et al., 2014).

### Notch Assays

Notch assays were carried out as described previously (Wagner et al., 2014).

### In Vitro Absorption, Distribution, Metabolism, Excretion, and Toxicity (ADMET)

#### Kinetic Solubility.

 BPN-15606 (100 *μ*M) was introduced into aqueous phosphate-buffered saline (PBS) buffer pH 7.4 as a concentrated solution in dimethylsulfoxide (DMSO) for 1 hour. The concentration of dissolved compound was measured via UV/Vis absorbance against a calibration curve.

#### Functional hERG.

An automated patch-clamp technique was used to measure hERG conductance using HEK293 cells stably transfected with the human ERG gene. Inhibition is expressed as an IC_50_ value determined from the percent inhibition versus test compound concentration curves using five concentrations of the test compound.

#### Liver Microsomal Stability.

BPN-15606 (1.0 *μ*M) was incubated with microsomes and cofactor regeneration system from human, rat, mouse, or dog for 30 minutes. Liquid chromatography (LC)/mass spectrometry (MS) analysis was then used to measure the remaining compound.

#### Microsomal Clearance.

The test compound (1.0 *μ*M) was incubated with liver microsomes and cofactor regeneration system from human, rat, mouse, or dog. LC/MS analysis was then used to measure remaining compound at specified time points (0, 5, 10, 15, 30, and 45 minutes).

####  Multi-Drug Resistance Gene 1-Madin Darby Canine Kidney (MDR1-MDCK) Permeability.

Passive and active transport of BPN-15606 across a monolayer of MDR1-MDCK cells in two directions (apical to basolateral and reverse) were evaluated. Permeability and efflux ratio of the test compound were determined based on measurement of compound concentration in the receiving compartment by LC/MS analysis.

#### CYP Inhibition.

The test compound was coincubated with CYP-selective probe substrates and human liver microsomes. Inhibition of the CYPs was assessed by measuring formation of metabolites of probe substrates by LC/tandem MS (MS/MS) analysis. Inhibition is expressed as an IC_50_ value determined from the percent inhibition versus test compound concentration curves using eight concentrations of the test compound. CYP isoforms evaluated include 1A2, 2B6, 2C8, 2C9, 2C19, 2D6, and 3A4 (two probe substrates are used for 3A4).

#### CYP Induction.

Test compound was incubated with cryopreserved plateable human hepatocytes from three individual donors. Upon incubation, CYP1A2, CYP2B6, and CYP3A4 enzyme activities are measured using specific probe substrates with LC/MS/MS detection to determine increase in enzyme activity compared with the vehicle control (fold induction). As positive controls, known CYP inducers are used. The acceptance criterion for assay performance (adequacy for detecting induction by BPN-15606) was a >twofold induction of CYP isoforms in the positive controls.

####  CEREP Profile.

 BPN-15606 (10 *μ*M) was incubated with a panel of 55 receptors (CEREP ExpresS, Redmond, WA) to determine potential off-target binding activity.

#### Metabolite Identification.

Test compound (1 *μ*M) was incubated at 37°C for 4 hours with cryopreserved mouse, rat, dog, monkey, or human hepatocytes. Identification of metabolites’ molecular weights and relative abundance of the specific metabolites was based on high performance liquid chromatography (HPLC)/MS/MS peak areas, and structural characterization of metabolites was accomplished by LC/MS/MS/Q-Trap.

### Bioanalytical Method

Mouse, rat, and nonhuman primate (NHP) plasma and brain extract calibration standards and quality control samples were prepared in untreated plasma or brain, as follows: A primary stock solution of 2 mg/ml analyte [BPN-15606 or *N*-(2-ethyl-2,4,5,6-tetrahydrocyclopenta[*c*]pyrazol-3-yl)-4-(6-methoxy-5-(4-methyl-1*H*-imidazol-1-yl)pyridin-2-yl)thiazol-2-amine (BPN-3783; internal standard)] in DMSO was prepared, and then dilutions were performed to make spiking solutions of analyte in DMSO at various concentrations. One volume of spiking solution was added to 99 volumes of untreated plasma or brain extract to attain nominal concentrations of standards with a final nonplasma matrix concentration of 1.0%.

#### Plasma Extraction Method.

To 50 *μ*l sample in a 2-ml microfuge tube was added 0.9 ml acetonitrile containing 1600 ng/ml BPN-3783 (internal standard). Tubes were vortexed at maximum speed on a plate vortexer 5 minutes and centrifuged 10 minutes at approximately 18,000*g*, and 20 *μ*l supernatant was removed to a glass HPLC vial containing 880 *μ*l 85/15/0.1:water/acetonitrile/formic acid (v/v/v).

#### Brain Extraction Method.

 Rat or mouse brain samples were stored frozen at −70°C and thawed to room temperature, and then homogenized in PBS using a probe sonicator (15 seconds at 50% maximal amplitude), using 4 ml PBS per 1 g brain tissue. To 50 *μ*l brain homogenate was added 0.3 ml acetonitrile containing 100 ng/ml BPN-3783 (internal standard). Tubes were vortexed, centrifuged 10 minutes at approximately 18,000*g*, and 75 *μ*l supernatant removed to a glass HPLC vial containing 800 *μ*l 85/15/0.1:water/acetonitrile/formic acid (v/v/v). The vials were briefly vortexed prior to LC/MS/MS analysis.

The ultraperformance LC/MS/MS method used a Shimadzu LC-20AD Pumps (Shimadzu, Columbia, MD), Leap Technologies CTC HTS PAL Autosampler (Leap Tecnologies, Durham, NC), a Phenomenex Luna C18(2), 50 × 2-mm, 5-*μ*m column (Phenomenex, Torrance, CA) operated at 20°C, and an AB Sciex QTrap 5500 mass spectrometer with multiple reaction monitoring (SCIEX, Concord, Ontario, Canada). The mobile phase consisted of (A) 0.1% formic acid in water (v/v) and (B) 0.1% formic acid in acetonitrile (v/v) delivered at 1.0 ml/min using a gradient elution mode. The initial elution condition was 2% B, which was maintained for 0.7 minute, increased to 95% B in 1 minute, and maintained for 1.7 minutes. It was then returned to 2% B in 0.2 minute and maintained for 1.5 minutes. The MS/MS analysis was performed using electrospray ionization, positive ion mode with the source temperature at 600°C. The ion spray voltage was 3500 V, and the collision energy was 40 eV for BPN-15606 and 45 eV for BPN-3783 (internal standard). Mass to charge ratios of 419.2 (precursor ion) and 297.1 (product ion) were used for multiple reaction monitoring of BMS-15606. Mass to charge ratios of 422.1 (precursor ion) and 214.05 (product ion) were used for multiple reaction monitoring of BPN-3783. Integration and quantitation were by Analyst software.

The lower limit of quantitation (LLOQ) was 1.0 ng/ml. The range of the plasma standard calibration curve (LLOQ) to upper limit of quantitation was 10.0 to 10,000 ng/ml. The coefficient of determination (r2) was ≥0.999 in the sample analysis.

### Animal Studies

All animal experiments were conducted following National Institutes of Health guidelines and were in compliance with the policies of University of California, San Diego Institutional Animal Care and Use Committee and the SRI International Institutional Animal Care and Use Committee (Palo Alto, CA).

### PK in Mouse and Rat

Male CD-1 mice and male Sprague–Dawley rats were administered a single dose of BPN-15606 by i.v. or oral gavage (po) dose routes to assess oral bioavailability. Mice and rats received BPN-15606 as a single i.v. dose at 1 mg/kg (*n* = 5) or a single po dose at 5 mg/kg (*n* = 5). Blood was collected from mice and rats at 5 (i.v. only), 15, and 30 minutes, and 1, 2, 4, 8, and 12 hours postdose for processing to plasma. For mice, brains were collected from both dose groups (i.v. and po) at 1, 4, 8, and 12 hours postdose. For rats, brains were collected from those same animals at the 12-hour time point only. Plasma, cerebrospinal fluid (CSF), and brains were collected at 1 hour postdose from another group of rats treated with BPN-15606 at 1 mg/kg (i.v.). All samples were analyzed by LC/MS/MS for BPN-15606 levels using a bioanalytical method that had a LLOQ of 1 ng/ml in plasma and CSF and 5 ng/g in brain tissue. Clinical observations were performed immediately postdose. All animals appeared normal throughout the study.

### PK Nonhuman Primates

Male non-naive cynomolgus monkeys (NHP) were administered BPN-15606 as a single nasogastric (po; 2 mg/kg) or i.v. (1 mg/kg) dose to assess oral bioavailability. Whole-blood samples were collected at specific time points and processed to plasma. Study in-life was conducted at Charles River Laboratories (Reno, NV). Pharmacokinetic (PK) plasma samples were shipped to SRI International for determination of drug concentrations. Samples were analyzed at SRI International using the bioanalytical method described above and validated for NHP plasma, and the LLOQ was 1 ng/ml.

### PK Analysis

Data were subjected to noncompartmental analysis using WinNonlin Phoenix Model 200 (for extravascular administration) or Model 201 (for i.v. bolus administration) and the sparse sampling feature; a uniform weighting factor was applied to each data set. Time to maximum plasma concentration (Tmax) and maximum plasma concentration (Cmax) values were determined directly from the data. Area under the plasma concentration–time curve to the last time point (AUClast) values were calculated using the log/linear trapezoidal (i.v. dose) or linear up/log down trapezoidal (po dose). Values were calculated using the plasma data from composite groups. The dose administered was input to the program as mg/kg, and as a result no additional corrections for individual body weights of the animals were necessary. The following parameters and constants were determined for the i.v. and po groups: observed Cmax, Tmax, AUClast, area under the plasma concentration–time curve extrapolated to infinity, terminal phase elimination half-life, and mean residence time to the last time point. The volume of distribution at steady state and clearance were calculated using the i.v. group data. Bioavailability after oral administration was calculated using the AUClast values for both the i.v. and po groups.

### Repeat-Dose Efficacy Studies in Mice and Rats

Male C57BL/6J mice (*n* = 10/dose level) and male Sprague–Dawley rats (*n* = 14/dose level) were administered either vehicle (80% polyethylene glycol 400 v/v; 20% sterile water v/v and 0.1% Tween 20 v/v) or BPN-15606 (10, 25, and 50 mg/kg) once daily po for 7 consecutive days (mice) or 5, 25, and 50 mg/kg once daily po for 9 consecutive days (rats). Clinical observations were performed immediately following dosing on each day, and all animals appeared normal throughout the study. Animals were sacrificed 4 hours following the dosing on day 7 of the 7-day oral treatment course (mice) and on day 9 of the 9-day oral treatment course (rats). Plasma and brain extracts were prepared (mice and rats); CSF was aspirated from the cisterna magnum with minimal to no blood contamination (rats only); and tissues were frozen at −70°C. A*β* peptides were quantitated in brain extracts and plasma (mice) or CSF and plasma (rats), as described previously, using Meso Scale multiplex kits and the Meso Scale Sector Imager 6000 (Kounnas et al., 2010). Statistical analysis was performed using GraphPad Prism software, and results are expressed as mean ± S.E.M. Analysis of variance was used to detect a significant effect. Drug levels of BPN-15606 were measured in plasma and brain extracts using the bioanalytical methods detailed above.

### Acute Single-Dose Pharmacodynamics in Mice

Male C57BL/6J mice (*n* = 5 per group) were administered a single dose of either vehicle (80% polyethylene glycol 400 v/v; 20% sterile water v/v and 0.1% Tween 20 v/v) or BPN-15606 (25 mg/kg) by po and sacrificed at 0.5, 1, 2, 4, 8, 12, and 24 hours following the dosing. Plasma and brain extracts were prepared, and A*β* peptides were quantitated as described previously (Kounnas et al., 2010). Statistical analysis was performed as described above for the repeat dosing studies.

### Chronic Repeat-Dose Efficacy in Transgenic Mice

Thirty-five female double-transgenic mice, model presenilin amyloid precursor protein (Jankowsky et al., 2004), purchased from The Jackson Laboratory (Bar Harbor, ME) [B6.C3-Tg (APPswe, PSEN1dE9) 85Dbo/Mmjax, stock no. 0034829] and then bred in-house, were used in the study at 90 days of age. The mice were housed in a temperature-controlled room at a constant 22°C in a 12:12-hour light/dark cycle (lights off at 18:00), with food and water available ad libitum. Age-matched mice were housed by treatment group in groups of two to four per cage. All experimental procedures were reviewed and approved by Institutional Animal Care and Use Committee at University of California, San Diego. Subjects were randomly assigned to either the drug group (BPN-15606) or vehicle, with individual groups ranging from 9 to 10 mice. Mice were treated for 6 months with BPN-15606 at a dose of 10 mg/kg/d. For the drug administration, BPN-15606 was milled into standard rodent chow, processed by Research Diets (New Brunswick, NJ). All animals were weighed three times weekly to assess any adverse effects on normal weight gain during the 6-month treatment period. Food consumption was determined by weighing the metal cage, including the chow, to the nearest 0.1 g.

#### Thioflavin S Staining and Amyloid Fibril Quantification.

To determine A*β* fibril plaque load, floating coronal sections from AD and wild-type mice, *n* = 4 for each group, were washed in miliQ water and mounted on Fisherbrand Superfrost Plus microscope slides, before being processed for 1% (w/v) Thioflavin S staining. All images were taken with Leica (Mannheim, Germany) fluorescence microscope at 5× and with the same exposure across all images. The percent plaque within specified areas of a series of hippocampal and frontal cortex sections was quantified using ImageJ software from National Institutes of Health. Images were converted to 8-bit grayscale, and area of interest was traced and determined. Brightness and B/W threshold were adjusted appropriately and consistently across all images. The area of plaque particles within the area of interest was obtained, and the percent plaque was calculated. The series of sections of each animal was averaged and grouped accordingly prior to statistical analysis. The number of series of section per animal ranged from three to 14 sections.

### Three-Dimensional Human Neural Cell Culture Model of AD

#### Cells and Drug Treatments.

HReN-mGAP AD ReN cell expression APP*^Swedish/London^* and Presenilin 1^ΔE9^ were previously described (Choi et al., 2014). The HReN-mGAP AD ReN cells were three-dimensional (3D)–differentiated for 7 weeks, as previously described (Choi et al., 2014). The cultures were then harvested and analyzed for the soluble (conditioned media) and insoluble A*β*40/42/38 and p-tau/tau levels, using Meso Scale Discovery electrochemiluminescence system. BPN-15606 (70 nM) and the same volume of DMSO controls were treated in the last 4 weeks. The potential toxicity of the drugs was monitored by CytoTox-ONE (lactate dehydrogenase) assay after 1 week of the treatments. At the end of the treatments, the soluble (media) and the insoluble fractions (5 M GuHCl-soluble extracts) were collected and analyzed for A*β*38, A*β*40, and A*β*42 with MSD enzyme-linked immunosorbent assay (ELISA; V-Plex 6E10 A*β*40/A*β*42/A*β*38 kit). The custom-made plates were used for detecting total tau (BT2; ThermoFisher Scientific, Sommerset, NJ) and pThr181 tau (At270; ThermoFisher Scientific, Sommerset, NJ). The insoluble fraction was dissolved in 5 M GuHCl, and pThr181 tau, total tau, A*β*40, A*β*42, and A*β*38 concentrations were determined by MSD ELISA. The levels of the control protein Tuj1 were determined by dot-blot analysis in the GuHCl lysates (data not shown).

### Repeat-Dose Toxicity and Micronucleus Evaluation of BPN-15606 in Rats

The objectives of this toxicity study were to determine maximum tolerated dose (MTD), characterize the potential toxicity, and calculate toxicokinetic parameters of BPN-15606 in adult male Sprague–Dawley rats (8–10 weeks of age) following daily po dose administration for 7 consecutive days, and to evaluate its potential to damage chromosomes or cause mitotic spindle abnormalities in vivo, as measured by the incidence of micronucleus formation in RNA-containing erythrocytes (bone marrow micronucleus assay). An overview of the study design is presented in Supplemental Table 8. Male rats were administered BPN-15606 po for 7 consecutive days at 30, 100, or 300 mg/kg (groups 2, 3, and 4). A control group (group 1) was given vehicle, 15% Labrasol/85% sterile water, at an equivalent dose volume (10 ml/kg). A 375 mg/kg dose (group 5) was included for determination of day 1 plasma drug levels. Clinical observations were recorded twice daily.

All rats in groups 1, 2, and 3 (0, 30, and 100 mg/kg, respectively) survived until their scheduled day 8 sacrifice, and all tissues specified for histologic evaluation were collected. None of the group 4 (300 mg/kg) animals survived the 7-day dosing period: three were sacrificed in moribund condition, and two were found dead. Necropsy examinations were performed on all rats, but, for group 4 animals, tissues for histopathologic examination were collected only from those rats that were sacrificed in moribund condition. Histopathology assessment was conducted on all tissues collected from all animals in groups 1 and 3, and all tissues collected from group 4 animals euthanized moribund. From group 2 rats, the following tissues were examined histopathologically: duodenum; jejunum; ileum; liver; lung; pancreas; spleen; stomach; and thymus, which were evaluated based on findings at the higher dose levels. When present, grossly altered tissues were examined histopathologically from all group 1, 2, and 3 rats, and from moribund sacrifice group 4 rats. Microscopic data were recorded, and a four-step grading system (minimal, mild, moderate, and marked) was used to define gradable changes. Terminology for data capture was consistent with International Harmonization of Nomenclature and Diagnostic Criteria as promulgated by the Society of Toxicologic Pathology.

The micronucleus component of this study was performed to evaluate the ability of BPN-15606 to induce chromosomal damage in bone marrow of male Sprague–Dawley rats. Rats (*n* = 5/group) were scheduled to receive single daily doses of BPN-15606 for 7 days at 30, 100, and 300 mg/kg/d (groups 2, 3, and 4, respectively), with euthanasia scheduled 24 hours after the final dose. Animals receiving 300 mg/kg/d were sacrificed in moribund condition on day 3 or 4 due to excessive toxicity, and micronucleus formation was not evaluated; all other treated animals survived to necropsy. Bone marrow samples collected from the low and mid dose groups (30 and 100 mg/kg) were evaluated for cytotoxicity and for micronucleus formation (see Supplemental Table 8). Three bone marrow smear slides were prepared per animal and fixed in absolute methanol. Positive control (cyclophosphamide) slides generated in a separate good laboratory practice (GLP)-compliant study were stained and included for scoring. Two slides per animal were stained with acridine orange, coded, and evaluated (blind) using epifluorescence microscopy. Under these conditions, the cytoplasm of polychromatic erythrocytes (PCE) exhibits orange fluorescence, and the DNA of any micronuclei present exhibits yellow fluorescence. The criteria employed for identifying and quantitating micronuclei are those described by Schmid (1975). Two principal parameters were determined, as follows: 1) the number of PCE among 200 total red blood cells (RBC) per animal (PCE/RBC ratio), which provides an index of bone marrow cytotoxicity, and 2) the number of micronucleated RNA-positive PCE among a total of 2000 PCE per animal, which provides an index of chromosomal damage.

### Dose Escalation/7-Day Oral Gavage Toxicity and Toxicokinetics Study of BPN-15606 in NHP

The objectives of this study were to determine the MTD of BPN-15606, characterize potential toxicity, and calculate toxicokinetic parameters of BPN-15606 in adult male and female cynomolgus macaques following daily po dose administration at a near-MTD dose of BPN-15606 for 7 consecutive days. In the dose escalation phase to establish the MTD (phase A), male and female cynomolgus macaques (*n* = 2/sex) were administered single doses of BPN-15606 at 3, 10, 30, 100, and 300 mg/kg, with 2 to 4 days between each dose escalation (Fig. 10). Clinical signs were limited to emesis in the 100 and 300 mg/kg groups. There were no apparent changes in the clinical pathology parameters after single-dose administration. The plasma drug concentrations at the approximate Tmax increased in a dose-dependent manner. Based on these results, 300 mg/kg was selected for evaluation in the 7-day repeat-dose phase of the study (phase B). Comprehensive evaluation of clinical pathology parameters (clinical chemistry, hematology), gross pathology, and histopathologic parameters was carried out at SRI International, according to their standardized protocols. Tissues examined histopathologically included the following: liver, lung, stomach, duodenum, colon, spleen, pancreas, thymus, heart, brain, and kidney.

## Results

### 

#### In Vitro Effects of BPN-15606 on *γ*-Secretase Activity.

**Fig. 1.**
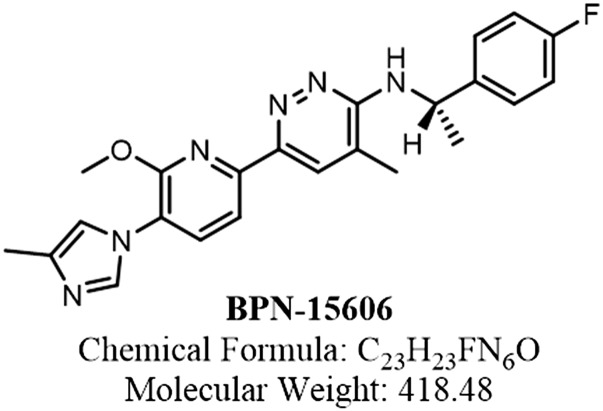
Chemical structure of BPN-15606.

**Fig. 2.**
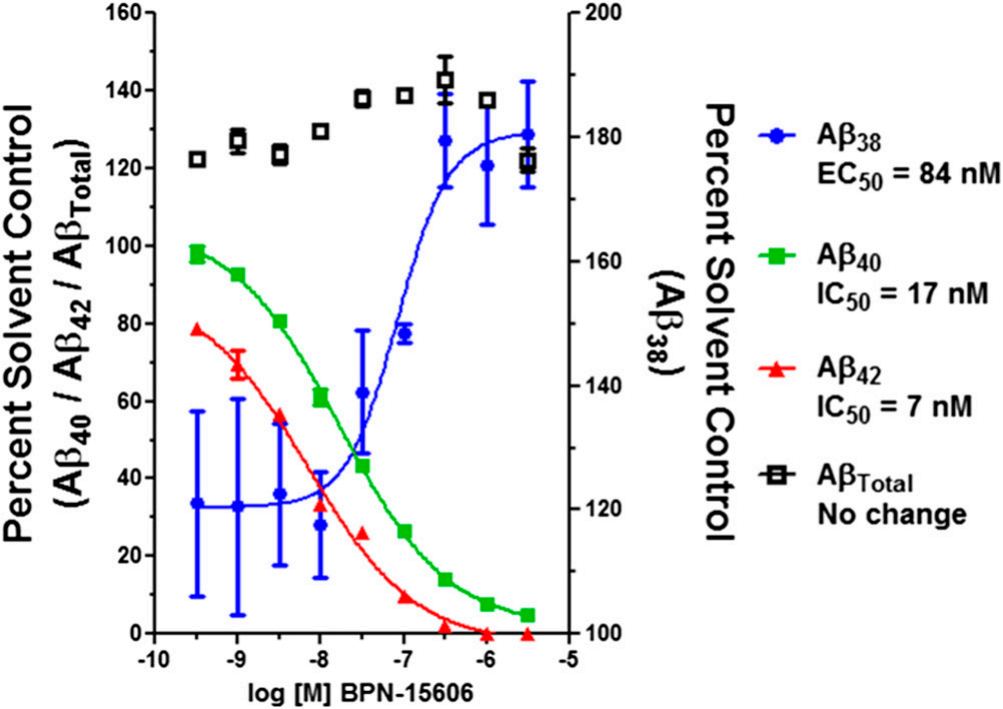
Concentration–response curves of BPN-15606 using SHSY5Y-APP cell-based MSD A*β* triplex screening assay.

**Fig. 3.**
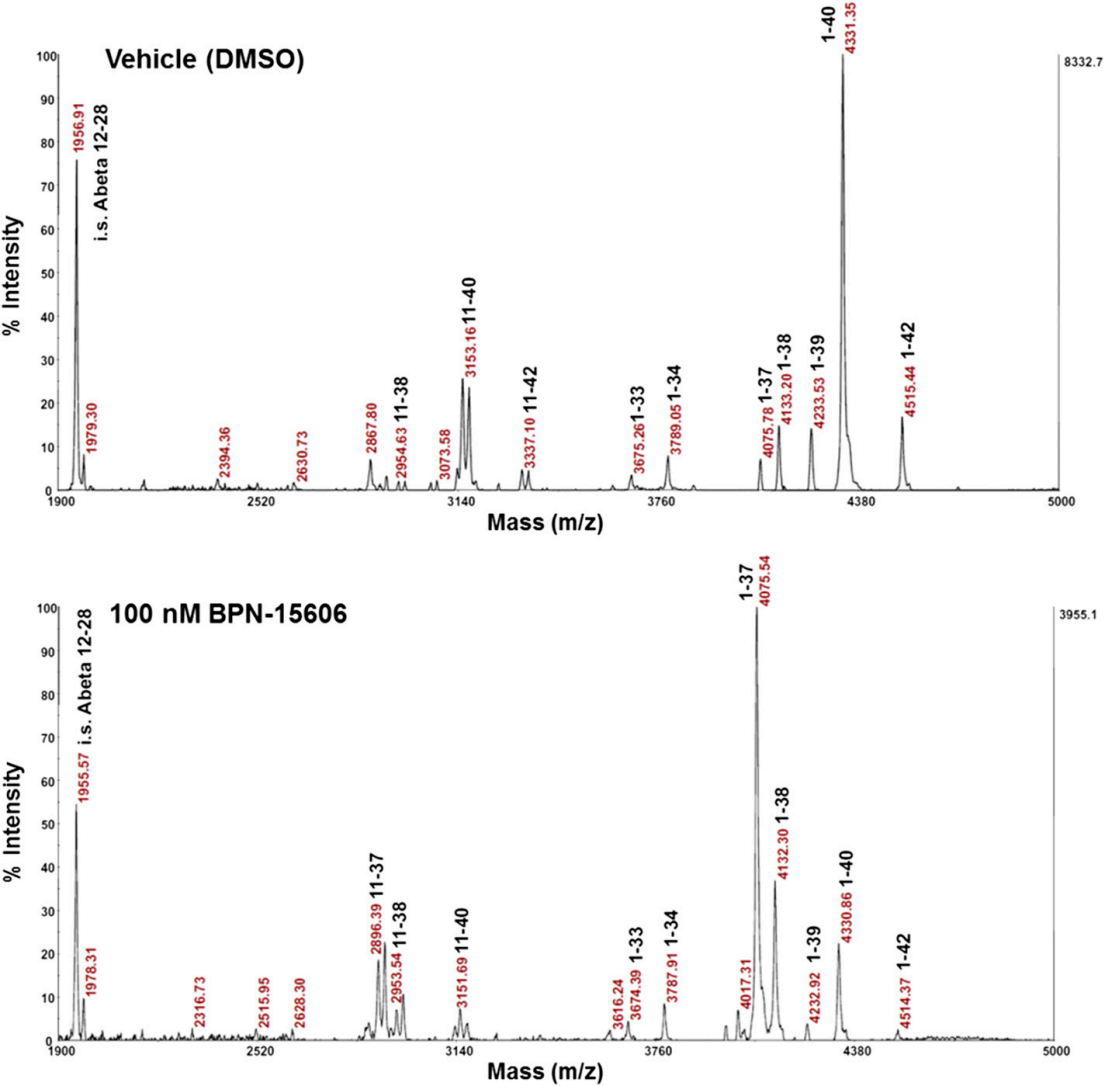
Incubation of BPN-15606 (lanes 1–6) with H4-APP751 neuroglioma cells transfected with the Myc-tagged Notch (NΔE) construct does not inhibit Notch proteolysis. Alternatively, incubation with *δ*-secretase inhibitor ***N*-[*N*-**(3,5-difluorophenacetyl)-*L*-alanyl]-*S*-phenylglycine t-butyl ester (lanes 7–10) potently inhibits Notch proteolysis.

During the past 4 years, we have synthesized and characterized over 600 novel GSMs encompassing four closely related scaffolds. Rigorous in vitro PK, pharmacodynamics (PD), and toxicological evaluation of these compounds led to the discovery of BPN-15606 (Fig. 1), which exhibits excellent drug-like properties. Based on the balance of data summarized in Supplemental Table 1, BPN-15606 progressed into nonclinical development. BPN-15606 is a GSM and, as such, binds to an allosteric site within the *γ*-secretase enzymatic complex and reduces the amount of secreted A*β*42 (Crump et al., 2013). In vitro, BPN-15606 exhibits an IC_50_ value of 7 nM for attenuating the production of A*β*42 by human SHSY5Y neuroblastoma cells stably overexpressing human APP751 wild-type as measured by ELISA (Fig. 2). Importantly, treatment with BPN-15606 does not affect Notch cleavage at doses as high as 25 *μ*M, as shown in Fig. 3. Proteolysis of Notch by *γ*-secretase is necessary for proper cellular differentiation, and inhibition of Notch cleavage by *γ*-secretase inhibitors was previously associated with significant side effects (Doody et al., 2013). Supplemental Tables 1 and 2 summarize the in vitro absorption, distribution, metabolism, excretion, and toxicity (ADMET) properties of BPN-15606 (Supplemental Table 1) as well as the in vitro metabolites identified in hepatocyte cultures from five species (Supplemental Table 2). Collectively, these studies established that BPN-15605 has an acceptable in vitro ADMET profile to enable in vivo studies and that there were no uniquely human metabolites identified.

**Fig. 4.**
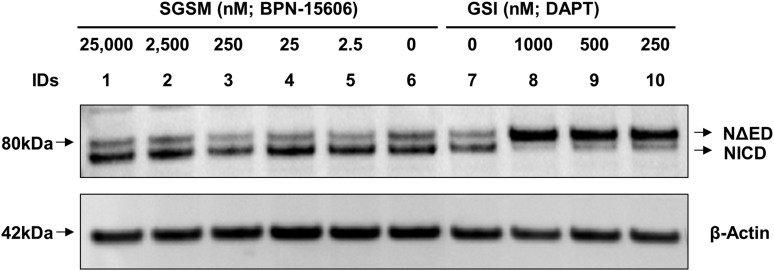
Matrix-assisted laser desorption/ionization time of flight mass spectrum data of anti–A*β*17–24 monoclonal antibody (4G8) immunoprecipitates.

This GSM mechanism is illustrated in the anti-A*β* immunoprecipitation/matrix-assisted laser desorption/ionization time of flight experiment depicted in Fig. 4, which shows that neuronal cells treated overnight with 100 nM BPN-15606 completely eliminate the peak corresponding to A*β*42 and significantly reduce the A*β*40 peak, whereas the size of the A*β*38 and A*β*37 peaks is significantly increased. This GSM mechanism appears to be free of the known toxicities associated with inhibiting the *γ*-secretase enzymatic complex that is responsible for cleaving a large number of type 1 membrane proteins (Wakabayashi and De Strooper, 2008; Haapasalo and Kovacs, 2011).

#### PK of BPN-15606 in Mice, Rats, and NHP.

Plasma concentrations of BPN-15606 measured in CD-1 mice and Sprague–Dawley rats were used to determine PK parameters, which are summarized in Supplemental Tables 3 and 4. Both species showed bioavailability of greater than 60%. In male non-naive cynomolgus monkeys (NHPs), plasma concentrations of BPN-15606 were readily quantitated, facilitating assessment of plasma PK parameters, which are summarized in Supplemental Table 5. These data indicate bioavailability is significantly less favorable relative to rodents (bioavailability of 30.8% compared with mice and rats, which were both greater than 60%).

#### Biochemical Efficacy of BPN-15606 in Mice and Rats.

**Fig. 5.**
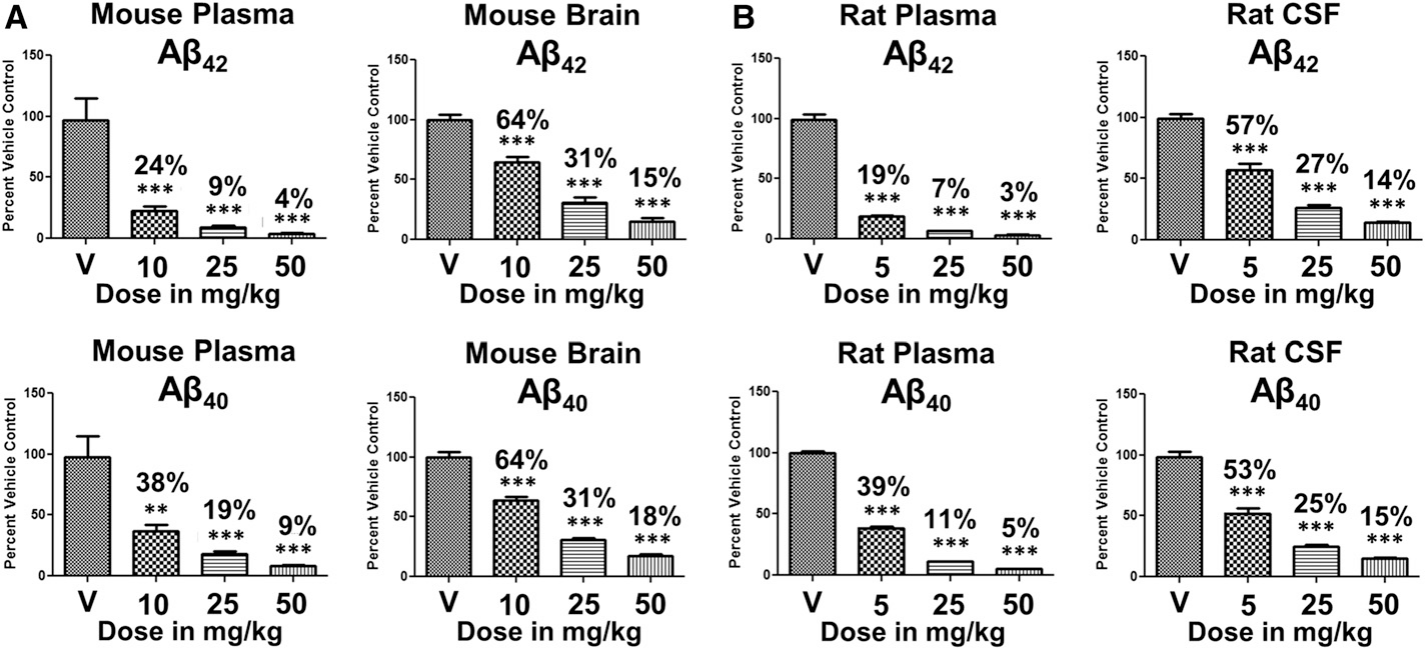
Levels of A*β*42 (top) and A*β*40 (bottom) in plasma, brain, or CSF following daily oral administration of either vehicle or BPN-15606. (A) A*β* peptides of male C57BL/6J mice (*n* = 10/dose) were measured in plasma and brain following a 7-day oral treatment course. (B) A*β* peptides of male Sprague–Dawley rats (*n* = 14/dose) were measured in plasma and CSF following a 9-day treatment course. A*β* peptide levels were determined using Meso Scale Sector 6000 Multiplex assays. Student t test; *P, 0.05; **P, 0.01; ***P, 0.001.

**Fig. 6.**
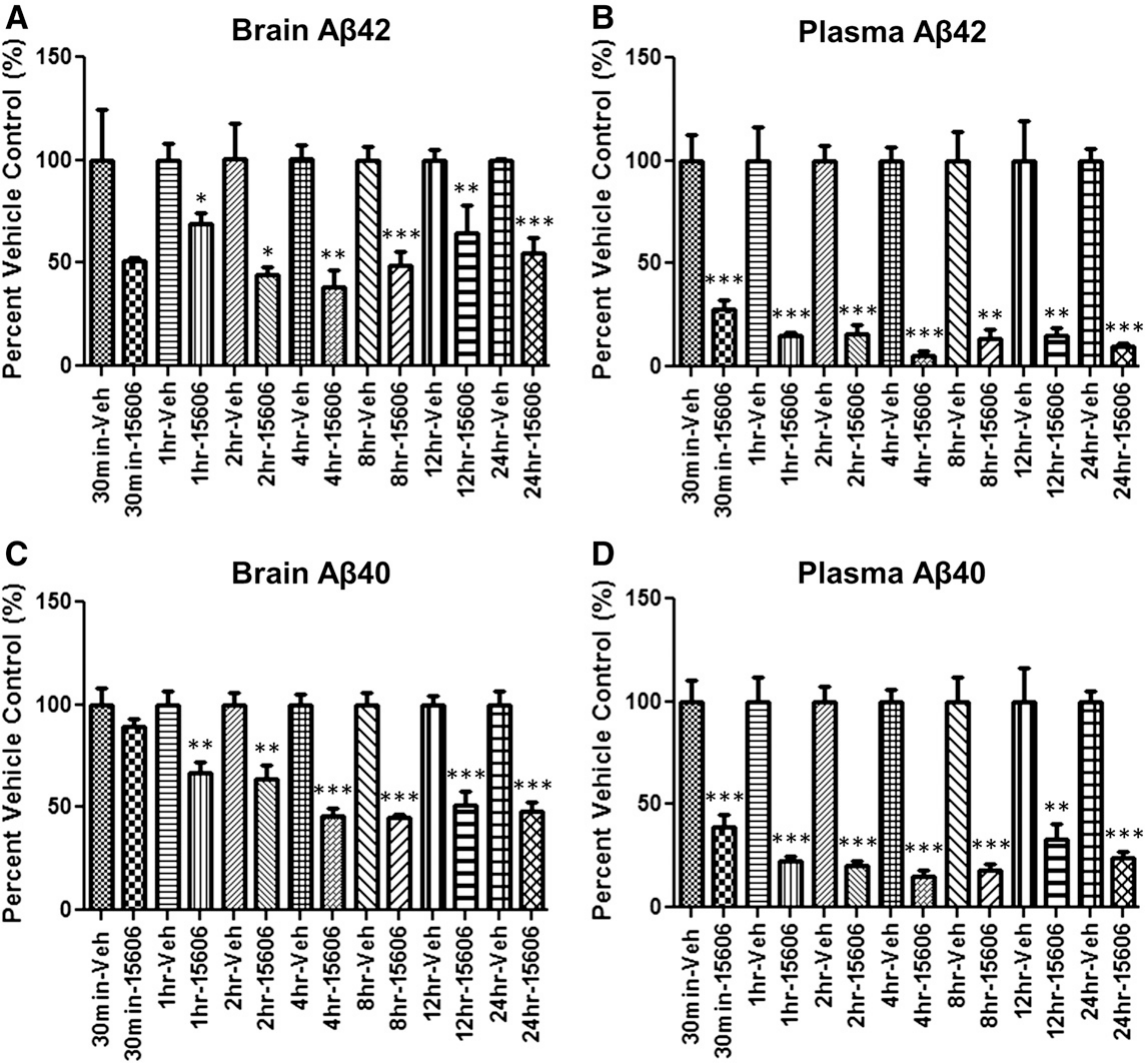
Levels of A*β*42 (A and B) and A*β*40 (C and D) in plasma and brain or following single oral administration of either vehicle or BPN-15606. C57BL/6 mice (*n* = 5/time point) were dosed with 25 mg/kg BPN-15606 or vehicle po. Animals were then sacrificed at the indicated time point (0.5–24 hours), and the levels of A*β*42 and A*β*40 peptides were quantified using Meso Scale Sector 6000 Multiplex assays. Student t test; *P, 0.05; **P, 0.01; ***P, 0.001.

BPN-15606 was repeatedly administered once daily po to both mice (7 days) and rats (9 days) at various doses (see Fig. 5). BPN-15606 showed excellent dose-dependent efficacy in both plasma and brain (mice) and plasma and CSF (rats) on lowering of A*β*42 and A*β*40 levels. Importantly, BPN-15606 also showed dose-dependent exposures in these two studies (see Supplemental Tables 6 and 7 for plasma drug levels in the same animals, in which the A*β* peptide levels were measured). The data in Fig. 5 also demonstrate excellent PD concordance between plasma and brain (mouse) and plasma and CSF (rat) for the ability of BPN-15606 to lower A*β*42 and A*β*40 levels in a dose-dependent manner. The effects of BPN-15606 were consistently greater in lowering plasma A*β* levels than either brain or CSF A*β* levels probably due to the higher exposures of free drug in plasma (see Supplemental Tables 6 and 7) as well as the shorter half-life of plasma A*β* peptides compared with the central nervous system turnover of A*β* peptides (Cirrito et al., 2003). Time course studies were also carried out in C57BL/6 mice following a single oral dose of BPN-15606 (25 mg/kg) (Fig. 6). As expected at this dose (25 mg/kg), BPN-15606 showed a robust effect on both brain and plasma A*β* 42 and A*β*40 levels, which began approximately 30–60 minutes following the single dose administration and lasted for ≥24 hours.

#### Efficacy of BPN-15606 in PSAPP Transgenic Mice Following Chronic Treatment.

**Fig. 7.**
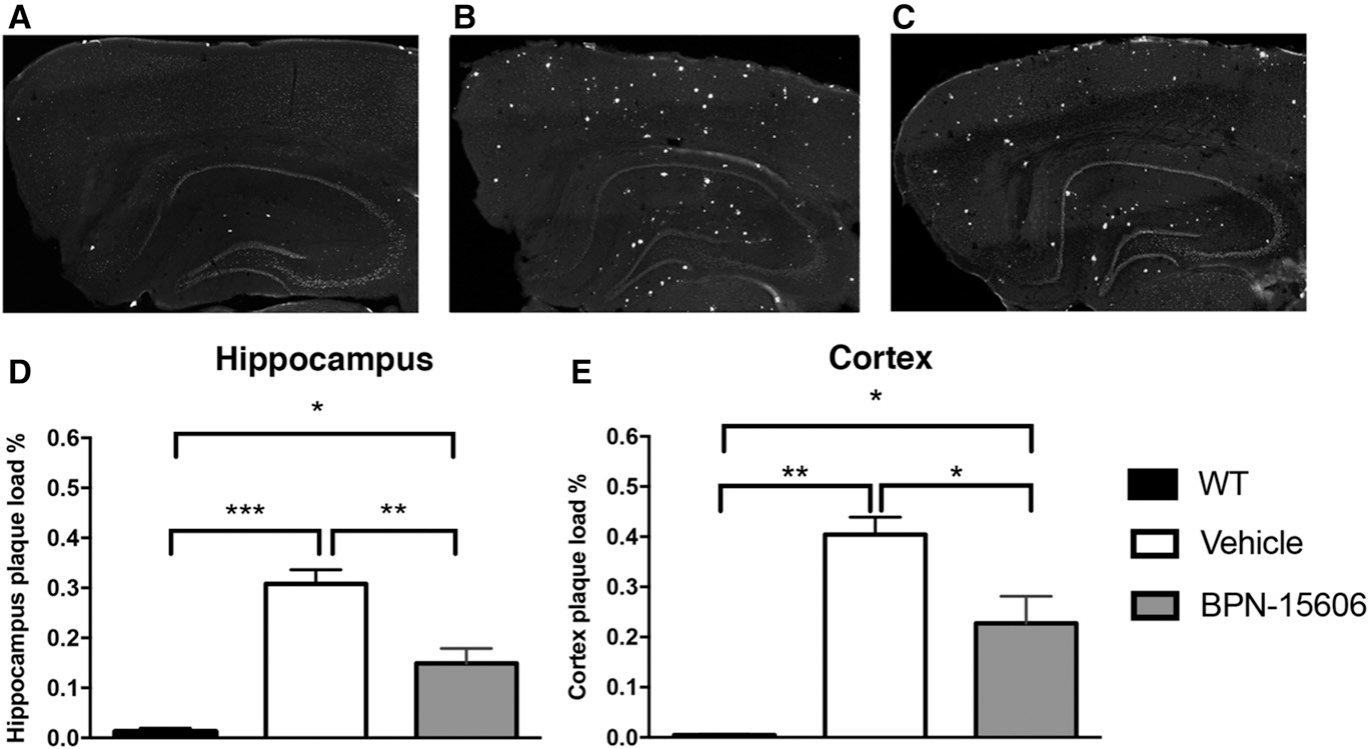
Representative coronal sections of Thioflavin S–stained images. Three-month-old PSAPP mice were treated with BPN-15606 (10 mg/kg/d) or vehicle via laced chow for 6 months. Grayscale images represent PSAPP mice at 9 months of age. (A) Representative coronal section of age-matched wild-type negative control littermates. (B) Representative coronal section of vehicle-treated PSAPP mice. (C) Representative coronal section of BPN-15606–treated PSAPP mice. A*β* accumulation in Thioflavin S–stained coronal sections was quantified using densitometry in 9-month-old PSAPP mice chronically treated for 6 months. BPN-15606–treated cohort had significantly reduced accumulation of A*β* plaques in both the hippocampus (D) and cortex (E). *n* = 10 per group. *P < 0.05; **P < 0.005; ***P < 0.0005.

To determine whether chronic BPN-15606 therapy could ameliorate A*β* plaque accumulation, the percentage of area occupied by A*β* plaques in PSAPP mice was quantified after 6 months of treatment with BPN-15606 at an estimated daily dose of 10 mg/kg. It is well established that the specific transgenic mouse model used in this study (PSAPP) develops A*β* deposits by 2–3 months of age with reliable onset of A*β* neuritic plaques at 6 months of age (Jankowsky et al., 2004; Zhang et al., 2016). Using Thioflavin S–stained coronal sections and densitometry, we confirmed that vehicle-treated female PSAPP mice at 9 months of age demonstrated significant accumulation of A*β* plaques (Fig. 7B) when compared with age-matched BPN-15606–treated PSAPP mice (Fig. 7C) and nontransgenic wild-type littermates (Fig. 7A). Regarding the quantitative analysis of treatment impact, densitometric measurements using NIH ImageJ software showed that chronic treatment with BPN-15606 significantly reduced accumulation of A*β* neuritic plaques in both the hippocampus and cortex (Fig. 7, D and E). Necropsies were carried out at the end of the study with no significant adverse findings in either the vehicle or BPN-15606 treatment groups (data not shown).

#### Efficacy of BPN-15606 on Soluble and Insoluble A*β*42, A*β*40, A*β*38, Total Tau, and p-Thr181 Tau in 3D Human Neural Cell Cultures.

**Fig. 8.**
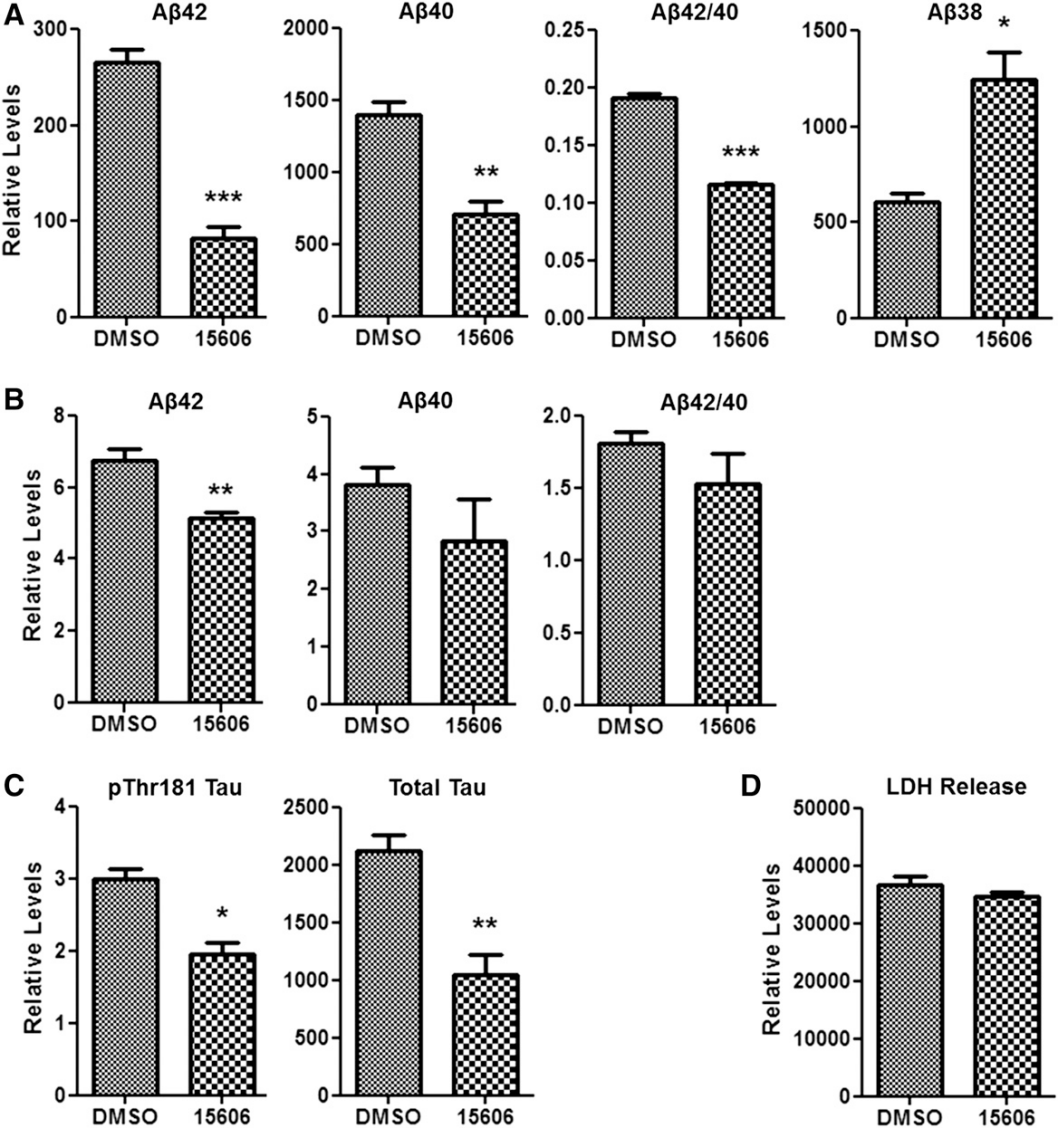
The impact of BPN-15606 on soluble and insoluble A*β*40/A*β*42/A*β*38 and p-tau/total-tau levels in 3D human neural cell culture model of AD. The AD ReN cells (HReN-mGAP30) were 3D-differentiated for a total of 7 weeks, whereas the 70 nM BPN-15606 and DMSO vehicle controls were treated for the last 4 weeks. (A) Relative levels of A*β*40, A*β*42, and A*β*38 in soluble (media) fractions. BPN-15606 treatment dramatically decreased A*β*42, A*β*40 levels, as well as A*β*42/40 ratio and increased A*β*38 levels. (B) Relative levels of A*β*40 and A*β*42 in insoluble (5 M GuHCl-soluble) fractions in the 3D-cultured cells with or without BPN-15606 treatment. (C) Relative levels of insoluble total and pThr181 tau. (D) The 1-week-old treated media were analyzed by the Cytotox cell death lactate dehydrogenase assay. BPN-15606 treatments did not induce any significant toxicity (*n* ≥ 4 per each sample). Student *t* test; *P < 0.05; **P < 0.01; ***P < 0.001.

BPN-15606 treatment (70 nM) dramatically decreased soluble levels of A*β*40, A*β*42, and A*β*42/40 ratio while increasing A*β*38 levels in the 3D human neural cell culture model of AD (Fig. 8A). BPN-15606 treatment also decreased insoluble (5 M GuHCl-soluble extracts) A*β*40 and A*β*42 levels (Fig. 8B). More importantly, BPN-15606 treatment decreased insoluble pThr181 tau and total tau levels (Fig. 8C), suggesting that BPN-15606 treatment reduces p-tau pathology as well as A*β* accumulation. This result is not unexpected because other A*β*42-lowering compounds (e.g., BACE inhibitors) have also been shown to impact p-tau pathology in this 3D human culture system (Choi et al., 2014). Interestingly, A*β*38 was not detected in insoluble fractions, suggesting that the A*β*38 species does not aggregate with A*β*40 or A*β*42 at least not in 3D human neural cell culture conditions.

#### Repeat-Dose Toxicity and Micronucleus Evaluation of BPN-15606 in Rats.

Animals in the vehicle- and 30 mg/kg BPN-15606–treated groups appeared normal throughout the treatment period. Several clinical observations were noted in the 100 and 300 mg/kg dose groups during the study, including hunched posture, hypoactivity, and discharge from nostrils, eyes, and/or mouth. All animals in the 300 mg/kg dose group were either found dead or sacrificed in moribund condition on day 3 or 4, indicating this dose exceeded the MTD. One animal in the 100 mg/kg toxicokinetic evaluation group died on day 4. This animal died within approximately 1 minute of administration of test article, and blood was present in the thoracic cavity on gross necropsy evaluation. Thus, the death was most likely due to procedural (gavage) error and not related to the BPN-15606. The primary hematology findings consisted of statistically significant, but small increases in RBC (↑10.5%), hemoglobin (↑10.9%), and mean corpuscular hemoglobin concentration (↑10.7%), and decrease in mean corpuscular hemoglobin (↓6.9%) that were present in rats administered 100 mg/kg (group 3). The absolute number and percent of reticulocytes were markedly decreased in the 100 mg/kg dose group, falling to about 20% of the control values after 7 days of treatment. Clinical chemistry changes were limited to small reductions in serum protein and albumin concentrations at 100 mg/kg (group 3), elevated serum cholesterol in both groups 2 (30 mg/kg) and 3, and lower triglycerides in group 3 compared with the controls; these changes were not toxicologically significant. Histopathologic lesions attributed directly (duodenum, stomach, liver) or indirectly (lung, pancreas, thymus) to BPN-15606 were limited to the mid (100 mg/kg)- and high (300 mg/kg)-dose groups, and were absent at the low dose (30 mg/kg). The duodenum displayed mucosal inflammation, edema, necrosis, erosion, ulceration, and/or serosal inflammation. Histopathologic changes in the stomach included the following: nonglandular epithelial hyperplasia, hyperkeratosis, erosion, and ulceration; nonglandular submucosal fibrosis, edema, increased inflammation, and fibrosis; and/or serosal inflammation. Hepatocyte basophilia, hypertrophy, and lipidosis were present in liver at the high (lethal) dose. Other microscopic observations in lung, pancreas, and thymus were secondary to malaise associated with gastrointestinal lesions.

BPN-15606 did not induce micronuclei in the bone marrow of rats following 7 days of treatment at 30 or 100 mg/kg, indicating it does not appear to have clastogenic potential in vivo.

**Fig. 9.**
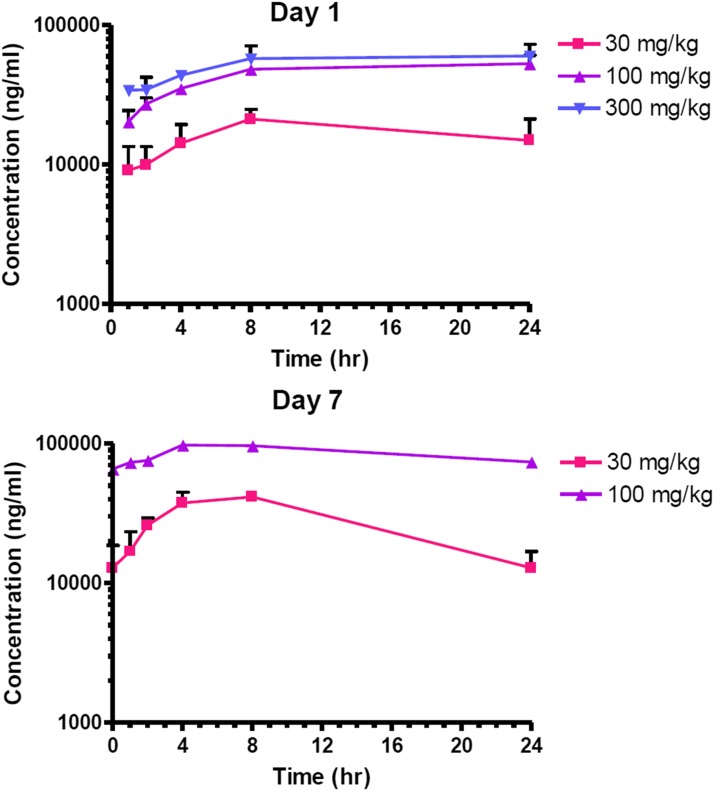
Plasma concentrations of BPN-15606 in male Sprague–Dawley rats on days 1 and 7. The test article (BPN-15606) was administered po daily for 7 days. All animals in the 300 mg/kg group were either found dead or euthanized on day 3 or 4.

BPN-15606 absorption was prolonged, with Tmax for individual animals occurring at 4–24 hours, depending on dose. The apparently continuous absorption of BPN-15606 from the gastrointestinal tract is most likely the result of slow dissolution rate caused by poor aqueous solubility. The mean Tmax was 13.3 hours (day 1) or 6.7 hours (day 7) in the 30 mg/kg dose group. The time to the peak plasma drug level was later on day 1 in the 100 and 300 dose groups, with a mean value of 18.7 hours. Mean values for Cmax and AUClast on day 1 increased with dose, although the increases were not proportional to dose from 100 to 300 mg/kg. The less than dose-proportional increase in systemic exposure (Cmax and area under the curve) of BPN-15606 is also most likely due to poor solubility. Day 7 predose samples all contained a significant concentration of BPN-15606, which resulted in exposure parameters that were markedly higher on day 7 than day 1 (see Fig. 9). The mean residence time to the last time point values ranged from 9.1 hours for the 30 mg/kg group on day 1 to 13.6 hours for the 100 mg/kg group on day 1. Thus, significant drug accumulation of BPN-15606 was observed in rats following once-daily dosing for 7 days.

In summary, BPN-15606–related mortalities occurred in the 300 mg/kg group. Adverse effects on the erythroid system are consistent with responses to acute hemorrhaging, which is supported by histopathologic findings of necrosis, erosion, and ulceration in stomach and duodenum. The liver was also identified as a target organ, based on microscopic evidence of basophilia, hypertrophy, and lipidosis in liver of rats treated with 300 mg/kg. There was no significant suppression of polychromatic erythrocytes (PCE) among RBC in bone marrow, and no statistically significant increase in the frequency of micronucleated PCE was seen at any dose level evaluated compared with vehicle controls. No toxicologically significant effects were observed in the 30 mg/kg dose group. The no-observed-effect level (NOEL) of BPN-15606 is thus 30 mg/kg/d when given po daily for 7 consecutive days to rats, and the MTD of BPN-15606 is considered to be approximately 100 mg/kg/d.

#### Dose Escalation/7-Day Oral Gavage Toxicity and Toxicokinetics Study of BPN-15606 in NHP.

**Fig. 10.**
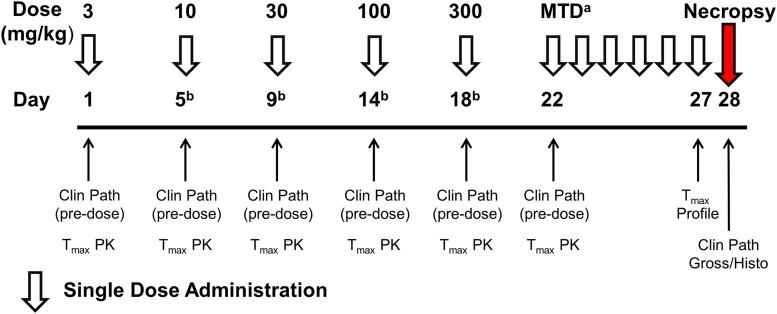
Design of NHP dose range-finding study. A dose escalation phase was used to determine the MTD that was followed by a 7-day repeat-dose phase at the MTD. Toxicokinetic time points: predose, 1, 2, 4 (T_max_), 8, and 24 hours. *^a^*Estimated based on clinical signs and clinical pathology in dose escalation. *^b^*Interval between doses was flexible and based on response/tollerance.

In the dose escalation phase, male and female cynomolgus macaques were administered single doses of BPN-15606 by the oral route at doses of 3, 10, 30, 100, and 300 mg/kg, with 2–4 days between each dose escalation (Fig. 10). Clinical signs were limited to emesis in the 100 and 300 mg/kg groups. There were no apparent changes in the clinical pathology parameters after single-dose administration for any of the doses evaluated. The plasma drug concentrations at the approximate Tmax increased in a dose-dependent manner. Based on these results, 300 mg/kg was selected for the 7-day repeat dose phase of the study (phase B). Daily oral administration of 300 mg/kg BPN-15606 resulted in clinical signs of emesis, reduced appetite, and reduced stool. Weight loss occurred with repeat-dose administration, which ranged from 3.2% to 10.5%. Based on the adverse clinical signs and weight loss, the study was terminated 1 day early (after 6 days of dosing).

**Fig. 11.**
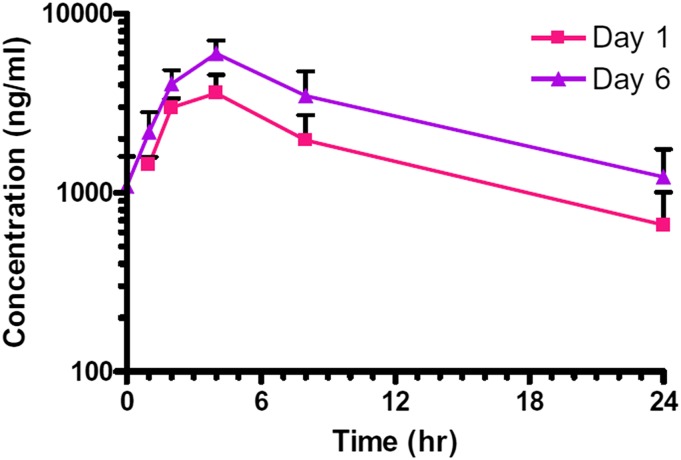
Plasma concentrations of BPN-15606 in male and female cynomolgus macaques on days 1 and 6 of the repeated-dose toxicology study. All predose plasma samples collected on day 6 contained measurable concentrations of BPN-15606. Each data point represents the mean ± S.D. of *n* = 4 monkeys (2 males and 2 females).

Toxicokinetic analysis of plasma drug levels identified a Tmax of 2–4 hours, and mean plasma half-life was 8.7 hours and 10.2 hours, on days 1 and 6, respectively. Daily administration of BPN-15606 led to accumulation, and, based on the AUClast, exposure to BPN-15606 was about 1.7-fold higher on day 6 than on day 1 (Fig. 11).

Histopathological findings that are considered to be related to repeated dose administration of BPN-15606 (300 mg/kg) were present in kidney and possibly liver. Renal findings included mild to moderate proximal tubular necrosis, casts, and dilation with cortical and medullary subacute inflammation in two male monkeys and one of two female monkeys. Hepatic findings were less convincing, but included minimal sinusoidal leukocytosis in two male monkeys and moderate focal fatty infiltration in one female monkey. These histopathological changes are consistent with the increased creatinine (renal effects) and increased total bilirubin (hepatic effects) that were noted in clinical chemistry analysis (data not shown). Adverse effects on the kidneys may be responsible for the decreased platelets and reticulocytes due to lower erythropoietin levels from kidneys and subsequent reduction in hematopoiesis. Changes in serum triglycerides and cholesterol are likely to be related to the observed weight loss, but may also be a direct effect of BPN-15606.

In summary, administration of a single dose of BPN-15606 to cynomolgus monkeys at doses of 3, 10, 30, 100, and 300 mg/kg resulted in emesis only at the 100 and 300 mg/kg dose levels. There were no other adverse findings noted during the single-dose administration phase. Based on these data, the MTD after a single-dose administration was estimated to be ≥300 mg/kg, and the NOEL after a single dose was 30 mg/kg. Administration of 300 mg/kg BPN-15606 po daily for 6 consecutive days to cynomolgus monkeys was associated with weight loss and adverse cortical and medullary renal findings, which were supported by changes in clinical chemistry parameters. Based on these observations and under the conditions of this study, the MTD is estimated to be significantly below 300 mg/kg/d. A no-observed-adverse-effect level (NOAEL) could not be determined for the repeat-dose range-finding phase (phase B).

## Discussion

We have shown in vivo PK/PD properties with BPN-15606, have characterized key toxicological properties of the molecule, and have demonstrated significant dose-dependent biochemical efficacy (lowering of CSF and brain A*β*42 levels by ∼40% at doses as low as 5–10 mg/kg in rats and mice, respectively; Fig. 5) and dose-proportional exposures from 5 to 50 mg/kg in both species (Supplemental Tables 6 and 7). At higher doses (25 mg/kg), BPN-15606 can almost totally eliminate (∼70–75% lowering) A*β*42 levels in both brain and CSF of mice and rats, respectively, whereas plasma A*β*42 is lowered by >90% in both species. These in vivo results are relevant because the CSF A*β*42 biomarker has been and is currently being used in clinical trials to assess target engagement (Fleisher et al., 2008; Coric et al., 2012; Lucey et al., 2015; Soares et al., 2016; Toyn et al., 2016).

BPN-15606 fulfills practically all of our lead identification profile criteria for a clinical candidate. Since the discovery of this pyridazine scaffold, further optimization efforts have been undertaken through synthesis of an additional 134 pyridazine GSMs, all of which are close structural analogs of BPN-15606 (Wagner et al., 2016) with the goal of identifying one or more backup compounds to BPN-15606 that are free of any significant liabilities. Furthermore, as we pursue a therapy for a challenging disorder such as AD, establishing a series of credible backup lead molecules to BPN-15606 is critically important.

We have previously shown that attenuation of A*β*42 levels over an extended period of time (7 months) dramatically reduces the number of amyloid plaques in Tg2576 transgenic mice (Kounnas et al., 2010). These data were generated following chronic oral treatment with 50 mg/kg/d aminothiazole-bridged aromatic GSM, or aminothiazole-bridged aromatic GSM (AGSM), similar in structure and function to the GSMs that we have been optimizing and characterizing over the past 4 years. The AGSMs act through a mechanism similar to our new lead GSM, by potently inhibiting A*β*42 and A*β*40 while potentiating A*β*38 and A*β*37.

### 

#### Biochemical and Pathologic Efficacy in Rodents and a 3D Human Neural Cell Model.

In this study, we have shown that a potent novel pyridazine C-ring–containing GSM (BPN-15606) significantly lowered A*β*42 levels in plasma and brain/CSF of mice and rats at doses 10-fold lower than doses previously required for AGSMs (Fig. 5) (Kounnas et al., 2010). In addition, BPN-15606 demonstrated dose-dependent efficacy in both mice and rats, as well as dose-dependent exposures at doses ranging from 10 to 50 mg/kg in mice and at doses ranging from 5 to 50 mg/kg in rats (Supplemental Tables 6 and 7). Importantly, the concentrations of free drug achieved in the brain at doses as low as 5–10 mg/kg in rats and mice, respectively, are still almost twofold above the in vitro IC_50_ for lowering A*β*42. The PK parameters of BPN-15606 in mouse and rat (Supplemental Tables 3 and 4) support the significant dose-dependent efficacies and dose-dependent exposures achieved in the two independent efficacy studies.

In the chronic (6-month) efficacy study in the PSAPP transgenic mice, treatment with BPN-15606 at a dose of 10 mg/kg/d starting with 3-month-old PSAPP mice, which presumably already had significant amyloid deposition, at 9 months of age, there resulted a substantial reduction in the amount of Thiofavin S–positive neuritic plaques in the BPN-15606–treated PSAPP mice compared with the vehicle-treated 9-month-old PSAPP mice (Fig. 7). These data demonstrating mitigation of neuropathology were consistent with the ability of BPN-15606 to substantially reduce the levels of insoluble A*β*42 as well as insoluble total tau and pThr181 tau in the 3D human neural cell culture model of AD, suggesting that BPN-15606 reduced p-tau pathology and A*β*42 accumulation (Fig. 8).

#### Repeat-Dose Toxicity.

In addition to potency, BPN-15606 has acceptable PK/PD properties, including bioavailability, half-life, and clearance (Supplemental Tables 3, 4, and 5). This PK/PD profile is reflected in the ability of the compound to sustain significant lowering of A*β*42 for ≥24 hours in mouse brain after a single dose (Fig. 6). BPN-15606 has undergone extensive preclinical study, including in vitro ADMET and metabolite profiling in five species (Supplemental Tables 1 and 2), which confirms the absence of any unique human metabolites. Additionally, this compound demonstrated a NOEL in the rat dose range-finding and toxicokinetic study of ≥30 mg/kg after 7 days of dosing (Supplemental Table 9) and a NOAEL of 50 mg/kg in the rat efficacy study after 9 days dosing in which significant efficacy was achieved at the lowest dose tested of 5 mg/kg (Fig. 5), suggesting a ≥10-fold safety margin based on dose. In this same rat study, the MTD was estimated at 100 mg/kg. BPN-15606 demonstrated a NOAEL of 30 mg/kg after a single dose in cynomolgus macaques, based on emesis noted at higher doses (Fig. 10; Supplemental Table 9). In the NHP dose range-finding and toxicokinetic study, a single-dose escalation phase was used to determine the MTD (300 mg/kg), which was followed by a repeat-dose phase of 6 days at the MTD. Emesis limited treatment duration to 6 days, which was accompanied by a weight loss of up to 10.5% relative to day 1, which was most likely related to emesis and reduced food consumption. There were no gross findings at necropsy, and histopathologic findings were limited to mild to moderate proximal tubular necrosis with subacute inflammation in two of two males and one of two females. In the liver, the histopathologic findings were reported as “less convincing” but included minimal sinusoidal leukocytosis in two of two males and moderate focal fatty infiltration in one of two females. As repeated oral administration of 300 mg/kg BPN-15606 to cynomolgus monkeys was associated with dose-limiting adverse effects after 6 days, doses of 100, 60, 30, and 10 mg/kg are planned for the Investigational New Drug-enabling 28-day GLP study.

#### Further Preclinical and Clinical Development.

To date no GSM has been tested in Alzheimer’s patients. In a series of excellent studies recently published by Bristol-Myers Squibb (Soares et al., 2016; Toyn et al., 2016), the GSM, BMS-932481, demonstrated robust translation across several preclinical species as well as human subjects. Unfortunately, in repeated oral dosing studies in humans, BMS-932481 elicited transient elevations in plasma concentrations of enzymes associated with hepatic toxicity, which precluded robust lowering A*β* peptides through oral dosing, thus terminating further clinical development of this once promising GSM. The authors suggested that cause of the elevation in hepatic enzymes by BMS-932481 was unlikely mechanism-based and possibly the result of the high degree of nonspecific protein binding that demanded plasma exposures in the micromolar range to achieve significant modulation of *γ*-secretase in brain (Toyn et al., 2016). Recently, another GSM (FRM-36143) was published by groups from McGill University (Montreal, Quebec, Canada) and FORUM Pharmaceuticals, which showed reasonable in vitro (EC_50_ = 35 nM) and in vivo potencies (maximum 58% reduction of A*β*42 in rat CSF at a dose of 30 mg/kg); however, the lack of any reported repeated exposure toxicity and safety data makes a prediction on the suitability of this compound for future clinical studies uncertain at this point (Blain et al., 2016).

Decisions on the future of BPN-15606 await the results of the Food and Drug Administration–reviewed planned 28-day GLP Investigational New Drug-enabling studies, although, based on the summation of the results of the studies presented on BPN-15606 in this work, in combination with traditional allometric scaling algorithms, we project that slightly less than micromolar plasma exposures will be necessary to elicit significant lowering of A*β*42 in human brain and should require much lower doses than those reported for BMS-932481 by Toyn et al. (2016) and Soares et al. (2016). Hopefully, BPN-15606 will ultimately be granted the opportunity to be tested in the appropriate human subjects and patients.

## Abbreviations

3D: three dimensional
A*β*: amyloid-*β* peptide
AD: Alzheimer’s disease
ADMET: absorption, distribution, metabolism, excretion, and toxicity
AGSM: aminothiazole-bridged aromatic GSM
APP: amyloid precursor protein
AUClast: area under the plasma concentration–time curve to the last time point
BACE: *β*-site amyloid precursor protein cleaving enzyme
BPN-15606: (*S*)-*N*-(1-(4-fluorophenyl)ethyl)-6-(6-methoxy-5-(4-methyl-1*H*-imidazol-1-yl)pyridin-2-yl)-4-methylpyridazin-3-amine
BPN-3783: *N*-(2-ethyl-2,4,5,6-tetrahydrocyclopenta[*c*]pyrazol-3-yl)-4-(6-methoxy-5-(4-methyl-1*H*-imidazol-1-yl)pyridin-2-yl)thiazol-2-amine
Cmax: maximum plasma concentration
CSF: cerebrospinal fluid
DMSO: dimethylsulfoxide
ELISA: enzyme-linked immunosorbent assay
GLP: good laboratory practice
GSM: *γ*-secretase modulator
HPLC: high performance liquid chromatography
LC: liquid chromatography
LLOQ: lower limit of quantification
MS: mass spectrometry
MS/MS: tandem MS
MTD: maximum tolerated dose
NHP: nonhuman primate
NOAEL: no-observed-adverse-effect level
NOEL: no-observed-effect level
PBS: phosphate-buffered saline
PCE: polychromatic erythrocyte
PD: pharmacodynamic
PK: pharmacokinetic
po: oral gavage
RBC: red blood cell
Tmax: time to maximum plasma concentration
